# Unveiling microsecond diffusion bonding phenomena enabled by air plasma spraying zirconia thermal barrier ceramics onto rare earth environmental barrier silicates

**DOI:** 10.1038/s41598-025-12218-w

**Published:** 2025-07-25

**Authors:** Edward J. Gildersleeve V, Emine Bakan, Marcin Rasinski, Moritz Kindelmann, Ashok Vayyala, Egbert Wessel, Joachim Mayer, Robert Vaßen

**Affiliations:** 1 Institute of Energy Materials and Devices, Materials Synthesis and Processing (IMD-2), Forschungszentrum Jülich GmbH, 52425 Jülich, Germany; 2 Institute of Fusion Energy and Nuclear Waste, Forschungszentrum Jülich GmbH, Management (IFN-1), 52425 Jülich, Germany; 3 Forschungszentrum Jülich GmbH, Ernst Ruska Zentrum (ERC), 52425 Jülich, Germany; 4 Central Facility for Electron Microscopy (GFE), RWTH Aachen University, 52074 Aachen, Germany; 5 institute of Energy Materials and Devices, Microstructure and Properties of Materials (IMD-1), Forschungszentrum Jülich GmbH, 52425 Jülich, Germany

**Keywords:** Air plasma spraying, Ceramic matrix composites (CMCs), Environmental barrier coatings (EBCs), Thermal barrier coatings (TBCs), Diffusion bonding, Aerospace engineering, Materials science, Structural materials, Techniques and instrumentation

## Abstract

While global propulsion and energy technology slowly progresses toward cleaner and renewable sources, gas turbine engines are still a workhorse infrastructure in these domains – for which achieving increased operating efficiency and reduced emissions is of significant importance. Reaching these ambitious goals would be enabled by the integration of more ceramic components in future turbines. Ceramic turbine components offer both reduced weight and reduced cooling needs, thereby benefitting turbine operating efficiency. However, these new components require robust and multifunctional surface coating solutions, which will be challenged by the future conditions of new turbines. Recently, a new class of multifunctional surface coatings achieved by layering state-of-the-art zirconia thermal barriers atop state-of-the-art rare earth disilicate environmental barriers has been shown. Despite their intrinsic thermomechanical incompatibility, these MultiLayered Thermal-Environmental Barrier Coatings (T-EBCs) have proven to offer several significant functionalities compared to their alternatives. Characterization limitations prevented the adequate description of the observed intrinsically-enhanced bonding between the zirconia and disilicate layer. In this work, advanced high-resolution characterization techniques are used to examine the T-EBC interface on the micro, nano, and atomic scale. A mechanism by which ceramic diffusion bonding can be achieved in-situ during fabrication of the layers is derived from the characterization results.

## Introduction

The global quest for cleaner and more efficient methods of energy generation and propulsion continues to drive innovations in materials and technology. While fully-renewable and/or carbon neutral technologies have yet to realize widescale global adoption, there is a growing demand and technological need to ‘bridge’ the gap in time. As an example, in aviation, efforts toward bridging the gap have been focused on improvements in gas turbine engine technology^1–5^.

While a well-established application that has been unilaterally adopted in both the aviation and power generation sectors, future gas turbine engines must demonstrate higher operating efficiencies and reduced emissions^6^. Optimizing both of these parameters simultaneously has proven to be one of the leading materials and engineering challenges over the last several decades. On the one hand, to reach maximum thermodynamic efficiency in a gas turbine engine, it is clear from the Joule-Brayton Cycle that the engine must run at ever-hotter firing temperatures^1,7–9^. Concurrently, however, when turbines run at hotter temperatures, the propensity for harmful NO_x_ emissions increases^6^.

To combat emissions, turbine manufacturers have several clear pathways of improvement. For instance, NO_x_ emissions can be reduced either by mixing alternative and more sustainable fuels (or injecting steam) into the combustion process or making structural changes to the engine to optimize airflow and reduce component cooling needs^6,10–12^. Contrastingly, achieving hotter operating temperatures can be nebulous. In lieu of hotter firing temperatures, the turbine community has focused on improving the temperature resistance of hot-section components (blades, combustors, vanes, etc.) – most notably via applying surface coatings. The original so-called Thermal Barrier Coatings (TBCs) are a prime example of this. TBCs have enabled the use of robust metallic nickel-based superalloy components at firing temperatures beyond their otherwise-uncoated temperature capabilities (i.e., achieving component surface temperatures $\:\ge\:$ 1200$\:^\circ\:$C with TBCs)^9,13–15^. However, as efficiency and firing temperature demands continue to increase, especially over the last decade, thermal limitations of TBC-coated superalloys are becoming a point of concern.

Specifically in the case of TBCs, the well-established material-of-choice 7 wt% yttria stabilized zirconia (7YSZ) has been observed over the last several decades of research to have distinct points of maximum temperature capability. At temperatures in excess of 1200$\:^\circ\:$C, over prolonged exposure, the advantageous metastable high-toughness t$\:{\prime\:}$ zirconia phase in 7YSZ decomposes into less favorable monoclinic and cubic phases^15–18^. Consequentially, 7YSZ TBCs in higher firing turbines are prone to experience infant mortality as compared to their anticipated durability. Solutions to enable still-higher firing temperatures for conventional Ni-based metallic turbine components have centered around layering a high-temperature-resistant oxide atop the 7YSZ material (i.e., Gd_2_Zr_2_O_7_) which protects the 7YSZ from the harmful degradation at high temperatures^19–21^.

Nonetheless, at present, especially in the aviation sector, there is a steady interest in overcoming these metallic component temperature limits by replacing Ni-based superalloy components with silicon carbide ceramic matrix composites (SiC-SiC CMCs)^9,22–24^. However, state-of-the-art SiC-SiC CMCs under turbine operating conditions will oxidize and form surface scales of SiO_2_, which then volatilize upon water vapor impingement^25,26^. The volatilization of the SiO_2_ scale can then accelerate the oxidation-volatilization process of the CMC, resulting in a gradual ‘thinning’ of the component. Under engine operating conditions, a SiC-SiC CMC in a turbine might experience recession rates on the order of several micrometers per hour, which is obviously technologically and economically untenable in long-term aviation applications^27,28^.

As it was in the TBC application, surface coatings are again at the forefront of turbine CMC research, albeit in this case the coatings are labeled as ‘Environmental Barriers’ (EBCs)^29–31^. To date, EBC research has seen several materials iterations; in general, they have converged around the use of silicate-based materials because of their inherent resistance to high-temperature water vapor corrosion. Modern EBCs are comprised of rare earth silicates, with the most water-vapor-resistant being the rare earth monosilicates (REMS) yet the most thermomechanically compatible with the SiC CMC components are the rare earth disilicates (REDS)^30^. In all coating cases (TBCs and EBCs), it is necessary to implement an intermediary coating (i.e., a ‘bond coat’) which can enhance the adhesive strength between the ceramic coating and the substrate material. In the case of EBCs for SiC-SiC CMCs, this bond coat is typically envisioned as pure silicon – which has a melting temperature of around 1400$\:^\circ\:$C.

One can then surmise, despite the implied temperature advantages of EBC-coated SiC-SiC CMCs and EBCs, the limitations of the Si bond coat could again inhibit CMC-based gas turbines from operating at the hottest, most efficient temperature conditions. Therefore, two combative strategies have been proposed in the literature: either replace the Si bond coat with something more temperature-resistant or reduce the temperature at the Si bond coat via through-thickness temperature control.

Si bond coat replacements have yet to be proven feasible in the literature, due to phase stability challenges and thermochemical interactions with the EBC layer at operating temperatures^32–35^. Yet al.ternatively, achieving the necessary through-thickness temperature drop to avoid melting (or softening/creep relaxation) of the Si bond coat is also nontrivial. The EBC must be sufficiently thick to offer the necessary temperature drop. Otherwise, equally detrimental in the context of operating efficiency and NO_x_ emissions, high component cooling fluxes are necessary. In either case, as the coating thickness increases or as the through-thickness temperature drop increases, the driving force for delamination, spallation, and premature failure of the EBC concomitantly increases^36–39^. To date, increasing the EBC thickness or utilizing increased component cooling are still the only strategies which have been proven feasible to implement. An alternative approach to this problem has been proposed in the past, wherein both TBCs and EBCs are integrated together in a single stack, forming a ‘Thermal-Environmental Barrier Coating’ (T-EBC)^1,29,40,41^. In principle, T-EBCs can achieve the desired temperature drops with an added advantage of doing so at overall lower coating thicknesses and with reduced component cooling requirements.

T-EBCs in the past literature generally utilized either highly complicated (i.e., 5 + component) thermal barrier oxides or expensive processing techniques – both of which offer non-negligible challenges toward scaling up to full industrial production levels^41,42^. Recently, we have shown that by using a conventional, well-established, low-cost deposition technique – Atmospheric Plasma Spraying (APS), it is possible to integrate readily-available low-cost zirconia-based TBC oxides (i.e., 7 wt% yttria stabilized zirconia, 7YSZ) with current state-of-the-art rare earth EBC silicates (i.e., Yb_2_Si_2_O_7_, or YbDS) to form novel T-EBCs^43^.

**Fig. 1:**
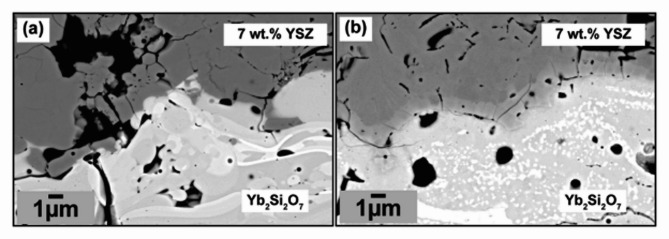
(**a**) Cross-sectional backscatter electron micrograph of the as-sprayed (or as-fabricated) zirconia/silicate T-EBC; the EBC layer is ytterbium disilicate Yb_2_Si_2_O_7_ (YbDS) and the TBC layer is comprised of 7 wt% Y_2_O_3_-ZrO_2_ (7YSZ). (**b**) shows the same coating after 200 hours’ heat treatment at 1300$\:^\circ\:$C.

Past works and fundamental mechanics point toward immense difficulty in plasma-spraying high-thermal-expansion-coefficient (CTE) thermal barrier oxides directly atop low-CTE silicate EBCs^43,44^. Yet the recent findings from our past work showed a surprising outcome, wherein a process pathway was found that allows the zirconia/silicate T-EBC to be thermo-mechanically stable (i.e., does not show macroscopic delamination of any kind) both after deposition and thermal cycling. This thermomechanical stability comes despite the large mismatch in CTE between the zirconia and underlying silicate/silicon/silicon carbide base material (i.e., 10–11 K^− 1^ vs. 5–7 K^− 1^, respectively). However, unexpected thermomechanical stability was only achieved in our work if the TBC layer was deposited atop an amorphous EBC layer. If the TBC was deposited atop a crystalline EBC, the same spallation challenges faced by others were observed^43,44^. Due to limited characterization techniques available at the time, the exact mechanism driving such an impressive interlayer bonding between the CTE-mismatched TBC and amorphous EBC was unclear. After 20-hour thermal cycling at 1300$\:^\circ\:$C for two hundred hours, an obvious reaction layer formed between the TBC and EBC, yet no significant delamination cracks or damage was observed at the interface; this is shown in Fig. 1b.

These first-of-their-kind results in the public T-EBC domain were encouraging toward the possibility of realizing industrially-applicable, fully plasma-sprayed T-EBCs – using commercially-available materials and straightforward processing conditions – as a scalable solution for the next-generation of SiC-SiC CMC turbine components. However, there are still several ambiguities which must be addressed to fully understand (and enable) the adoption of these high-risk zirconia/silicate coatings in the industrial sector. To that end, this work looks to utilize several high-resolution characterization techniques concurrently to address the following unanswered questions:

- What drives interlayer bonding between the 7YSZ TBC and YbDS EBC to be enhanced when the EBC layer is amorphous as opposed to crystalline prior to TBC deposition?
- How does the TBC material interact with the underlying EBC material during impact and rapid solidification processes that are characteristic to the APS process? Are there any physical or chemical changes that would drive a change in interlayer strength?
- Does the deposition process of the TBC layer change the crystallinity of the amorphous EBC layer underneath in any meaningful way?

## Experimental methods

### Thermal spray deposition

For this paper, a combination of existing samples from our past work and newly deposited samples were analyzed. For new depositions, it was necessary to use the established thermal spray processes from our past work^43,45,46^. All of the newly deposited samples here were specifically fabricated to deposit single splats of material. To achieve this, the plasma spray torch [F4MB, Oerlikon Metco, Wohlen, Switzerland] mounted to a six-axis robot arm [ABB IRB1600, ABB, Germany] was rastered in front of the sample surface at high speeds (in this case, 1250 mm s^− 1^) such that only very few droplets can deposit onto the surface. In all deposition cases, commercial-grade, readily-available thermal spray feedstock powder was used. Material selection was the same as in our past work^43^. For the TBC oxide, 7 wt% yttria stabilized zirconia (7YSZ) was used. For the EBC, ytterbium disilicate, Yb_2_Si_2_O_7_ (YbDS) was used. Additionally, prior to splat deposition, the specimens were preheated to similar deposition temperatures (~ 300-400$\:^\circ\:$ C) as in the fabrication of full-thickness coatings from our past work. Also prior to splat deposition, the sample surfaces were be pre-polished to a near-mirror finish (i.e., R_a_ $\:\le\:$ 1 μm). This was done by hand using standard metallographic methods. A summary of the thermal spray deposition parameters for the new single splat experiments is shown in Table 1.

**Table 1:** Plasma spray processing parameters used to deposit single splats for this study. The robot traverse speed was kept constant at 1250 mm s^-1^ for all deposition experiments.

Sample	Splat Material	Substrate	Feed Rate (gram min^− 1^)	Ar (slpm)	H_2_ / He^§^(slpm)	Current (Amps)	Standoff Distance (mm)
A	7YSZ	VA Steel	5	47.5	6	550	150
B	Yb_2_Si_2_O_7_	Si-coated Hexoloy™ SA SiC	5	46	4^§^	325	90
C-1	7YSZ	Yb_2_Si_2_O_7_ (Amorphous)	5	47.5	6	550	150
C-2	7YSZ	Yb_2_Si_2_O_7_ (Crystalline)	5	47.5	6	550	150

### FIB-SEM microstructural characterization

A dual beam device [Crossbeam 540, Carl Zeiss, Zeiss Inc, Germany] equipped with a focused ion beam (FIB) column was used to prepare cross-sections through the individual thermal spray splats. The cross-sectioning process was divided into two processes, each using a different Ga ion beam current. The initial trench was cut with 30 kV 7nA beam, and was then further cleaned with 30 kV, 1.5nA beam. The final prepared cross-sections were further analyzed using the Schottky field emission high resolution scanning electron microscope (SEM) in secondary electron (SE) and backscattered electron (BSE) imaging modes. SEM images for these purposes were recorded using either a 5 kV, 1nA electron beam or 12 kV, 2.5nA electron beam for SE and BSE mode, respectively. In some cases, images were taken at 1.6 kV, 800pA using an Energy selective Backscattered (EsB) detector. Chemical analysis in the form of line scans or mapping was done by Energy Dispersive X-ray Spectroscopy (EDX) employing a Silicon Drift Detector [X Max 80, Oxford Instruments, United Kingdom] with a 12 kV, 2.5nA electron beam.

### STEM microstructural characterization

To investigate the interface between the YSZ top layer and the amorphous disilicate layer, electron transparent lamellae were cut from a representative area of the fully-coated sample (as in Fig. 1a) at the TBC-EBC interface using focused ion beam milling methods [FEI Helios NanoLab 460-F1, USA]. Scanning transmission electron microscopy (STEM) was performed at 200 kV using a probe corrected TFS Spectra 300 microscope (Thermo Fischer Scientific, USA), which is equipped with a Super-X EDS detector.

### Laser assisted atom probe tomography (LAAPT)

In this study, APT ‘needles’ were prepared from the TBC-EBC interface using a standard FIB lift-out technique from the same sample as in the STEM experiments^47^. This FIB preparation was carried out using the same Helios Nanolab 650i dual beam FIB. The APT was operated in the laser mode, with laser pulse frequencies between 200–250 kHz and laser pulse energies ranging from 30–50 pJ. The detection rate was set to 0.005 ions per pulse. During the measurement, the temperature of the specimen was held at 50 K. The APT analysis was performed with a LEAP 4000X HR [Ametek Inc., USA]. For the 3D reconstruction and analysis of the APT data, the Integrated Visualization and Analysis Software (IVAS™) package, version 3.6.14 from Ametek Inc., was utilized.

### SEM-EDX-EBSD microstructural characterization

For the Electron Backscattered Diffraction (EBSD) analysis, epoxy-mounted specimen which were metallographically polished with a final finish step of 50 nm colloidal SiO_2_ were imaged in a field emission SEM [Merlin, Carl Zeiss, Oerzen, Germany] equipped with Oxford EDX and EBSD detectors [Oxford Instruments, Dresden, Germany].

## Results and discussion

### Baseline experiments to define the system and methodology for characterization and analysis

**Fig. 2:**
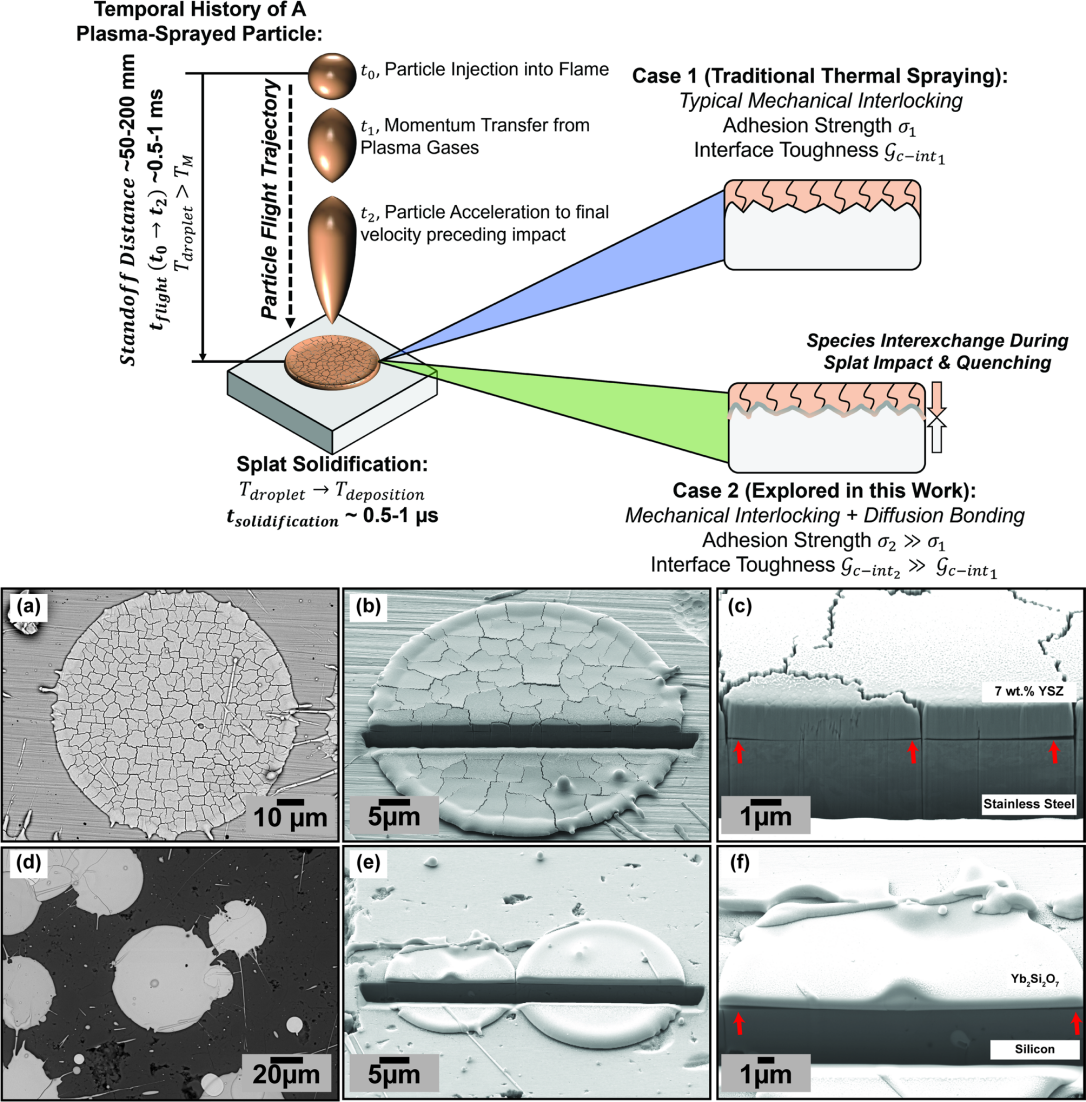
Schematic illustration of the Plasma Spray Deposition process for polycrystalline ceramic-based materials (i.e., zirconia thermal barrier oxides / TBCs) on metallic substrates. A representative backscatter electron image of a 7YSZ splat on stainless steel is shown in (**a**). Secondary Electron images of FIB cross-sections of an equivalent splat are shown in (**b**) and (**c**). For comparative purposes, splats of Yb_2_Si_2_O_7_ deposited atop silicon bond coats are shown in (**d**) – (**f**) to demonstrate this material does not undergo the same cracking/stress-relief mechanism. Red arrows in (**c**) and (**f**) indicate areas where the quenching splat material locally debonded from the planar surface due to stresses during cooling.

As explained in the introduction, the experimental efforts from this work seek to unveil the complex interactions and bonding mechanisms specific to the APS deposition of 7YSZ TBCs atop amorphous rare-earth YbDS EBCs. To try to understand why the TBCs deposited on amorphous EBCs from our past work have superior adherence, it is necessary to define and understand the system at these initial stages of TBC deposition. Figure 2 schematically defines the system which is being considered in this study, while also providing representative baseline FIB-SEM images to aid in the overview of terminologies. Briefly, in the APS process, molten (assumed fully-molten here) droplets of coating material will impact a warm (300-400$\:^\circ\:$ C) substrate, then rapidly solidify (on the order of 0.5–1 µs) to form single ‘splats’, as illustrated in the schematic^48,49^.

The successive buildup and deposition of several hundreds of these individual 0.5–1 μm thick (see Fig. 2c, f) splats will yield a 300–400 μm thick coating. For the classic case of zirconia-based TBC materials on metallic substrates, during quenching and solidification, the splat can face massive ($\:\ge\:$ 1 GPa) stresses as governed by the thermal strains during quenching, i.e., $\:{\sigma\:}_{splat}\approx\:{E}_{splat}{\alpha\:}_{splat}{\Delta\:}T$. Here, $\:{E}_{splat}$ is considered to be nearly the elastic modulus of the bulk material, and $\:{\alpha\:}_{splat}$ is its CTE^50,51^. Polycrystalline ceramics, such as zirconia-based TBCs, relieve these stresses by forming through-thickness channel cracks (usually referred to as ‘microcracks’). The microcracked network in TBC splats can be seen in Fig. 2a-c. In contrast, glassy ceramic materials which cannot crystallize during rapid quenching (i.e., Yb_2_Si_2_O_7_ and other silicate EBCs) will not form these through-thickness channel cracks in spite of their obvious brittleness relative to polycrystalline ceramics (see Fig. 2d-f)^45,52^.

For this study, it is also necessary to define the two potential bonding mechanisms in plasma-sprayed layers. In the traditional case of interlayer/intersplat bonding in APS, the quenched liquid, as it solidifies, should conform to and ‘interlock’ with the particular surface features/topography of the underlying substrate (termed ‘mechanical interlocking’, Case 1 in Fig. 2). In rare cases, when the local deposition temperatures are high enough, it can be possible for the molten impinging droplet/splat to ‘remelt’ the underlying material, leading to chemical interactions^53,54^. In such a scenario, there is an accompanied interexchange of atomic/ionic species between both splat and substrate and, in addition to the mechanical interlocking, a chemical diffusion bond can form (Case 2 in Fig. 2). If one assumes the adhesive strength (in MPa) and interfacial toughness (in J/m^2^) of the mechanical interlocking Case 1 bond to be $\:{\sigma\:}_{1}$ and $\:{\mathcal{G}}_{Ic-in{t}_{1}}$, respectively, then the corresponding strength and toughness of the diffusion-bond-enhanced Case 2 interface (notwithstanding the formation of brittle reaction products, i.e., in the case of some ceramic-metal diffusion-bonded structures) becomes $\:{\sigma\:}_{2}\gg\:{\sigma\:}_{1}$ and $\:{\mathcal{G}}_{Ic-in{t}_{2}}\gg\:{\mathcal{G}}_{Ic-in{t}_{1}}$, respectively^55–57^.

It is important to note here that the aforementioned diffusion bonding (during microsecond splat deposition) has only been seen in thermally-sprayed metals^58,59^. In the case of thermally-sprayed ceramics, there has yet to be any literature which has proved the propensity for a diffusion bond between two ceramic coatings to form due to (or during) the spray processing. Past work has shown the propensity for 7YSZ splats deposited on mirror polished stainless steel (i.e., the case of Fig. 2a-c) to promote Zr cation diffusion into the 20–30 nm thick Cr_2_O_3_ thermally grown oxide scale of the substrate^60^ – however this is fundamentally different from the concept posed here – where 7YSZ is being deposited onto an underlying glass-ceramic coating that cannot oxidize prior to splat impact.

Our past work suggested the possibility that the deposition of TBCs on amorphous EBCs does indeed qualitatively, yet unequivocally elicit stronger bonding than when sprayed onto crystalline EBCs^43^. Moreover, our past work’s results pointed toward the possibility that interexchange between the TBC and EBC ionic species truly is taking place. However, at the time, the results were inconclusive to definitively say the interface was indeed diffusion-bonded. The results that follow here will strive to use high-resolution characterization techniques to study and confirm whether species interexchange can occur upon deposition of the first TBC splats.

### Focused ion beam cross-sectioning of APS 7YSZ splats on Yb_2_Si_2_O_7_

As shown in Fig. 2, typical APS splats after solidification are between 10 and 100 μm in diameter and only 500 nm–1 μm in thickness. These length scales present challenges in utilizing traditional characterization methods to study the 7YSZ/YbDS interface. Understandably, ceramic splats are highly brittle and easily susceptible to damage by conventional metallographic and cross-sectioning techniques. It then becomes clear from this information and from Fig. 2 that FIB cross-sectioning of individual APS splats is the optimal method to view, characterize, and study the interface between the 7YSZ TBC and YbDS EBC material. The SEM images in Fig. 2a-f also intend to serve as a reference for the reader of the typical microstructures of APS splats of 7YSZ and YbDS in their traditional configurations. From the Figure, there are two key observations to extract from this reference case. First, the impact and solidification of molten ceramic material does not seem to influence the underlying topography of their substrates. One can clearly see the interface between the 7YSZ splat and stainless steel (as well as the YbDS splat and silicon) remains at its mirror-like polished state. Rather than deform the metal, the ceramic splats clearly undergo debonding during cooling due to CTE mismatch stresses (as indicated by the arrows in Fig. 2c and f). In addition, as described earlier, 7YSZ forms microcracks while quenched YbDS typically does not.

**Fig. 3:**
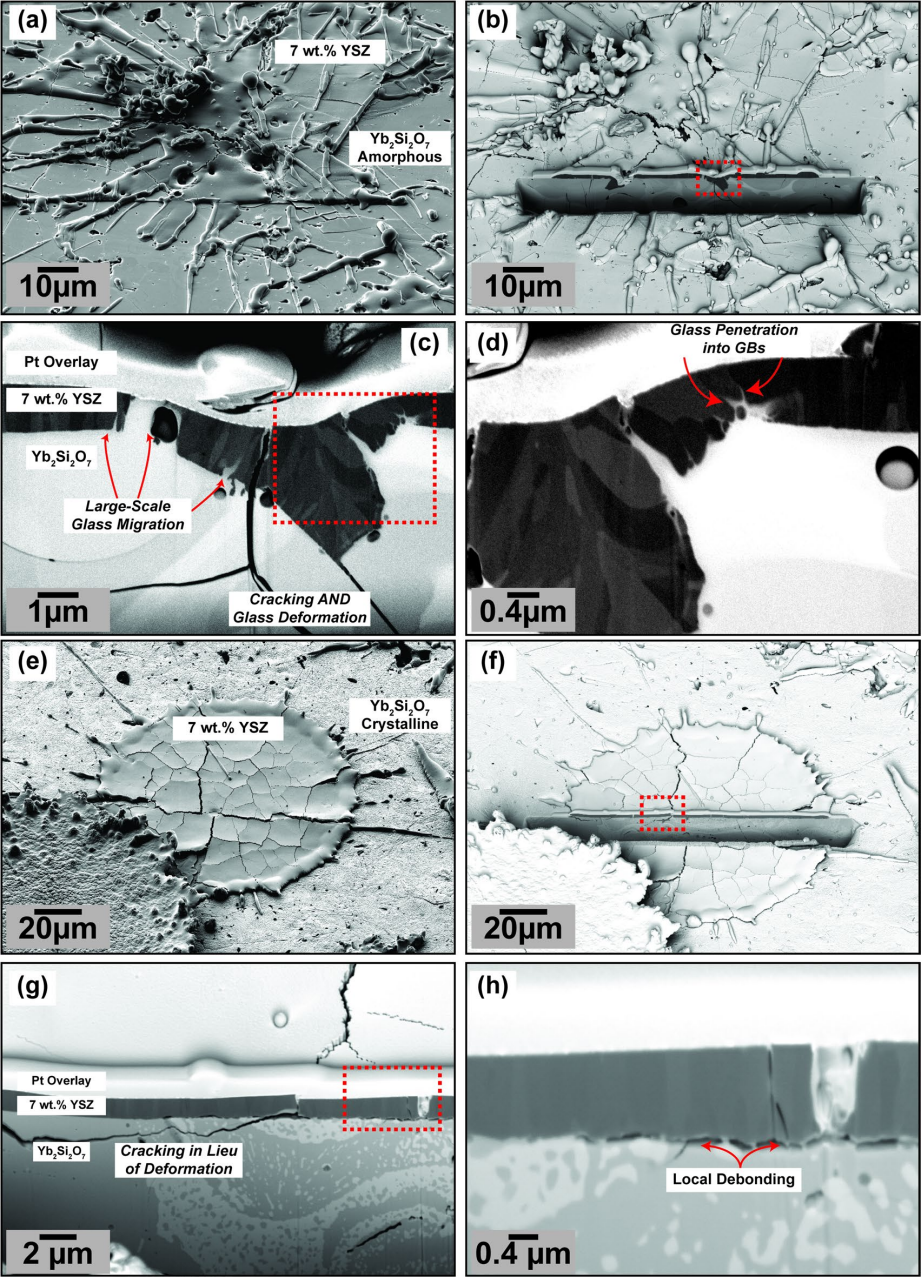
FIB cross-sections of plasma-sprayed 7YSZ single splats on two types of plasma-sprayed YbDS coatings. (**a-d**) show a 7YSZ splat deposited on amorphous YbDS, sample C-1 from Table 1. (**e-f**) show a 7YSZ splat deposited on crystalline YbDS, sample C-2 from Table 1.

Building off the reference baseline scenarios shown in Fig. 2, the next series of experiments conducted focused on depositing the same 7YSZ splats as in Fig. 2a-c, but in this case onto amorphous and crystalline YbDS coatings (samples C-1 and C-2 from Table 1, respectively). Figure 3 shows the resulting splats from these two deposition experiments and their respective FIB cross-sections. It is important to note here that the specimens from Fig. 3 were prepared under deposition conditions similar to both the T-EBCs in our past work and the T-EBC shown in Fig. 1^43^. From Fig. 3, there are several important findings.

#### Crystallinity-dependent YbDS deformation during 7YSZ impact and solidification

First, it is clear that the impinging molten (at temperatures > 2700$\:^\circ\:$C) 7YSZ droplet clearly deforms the originally planar, mirror-like surface of the amorphous YbDS material upon impact and solidification as seen in Fig. 3b-d. This becomes clear comparing the 7YSZ reference cases from Sample A (Fig. 2a-c) with Sample C-1 (Fig. 3b-d). It is important to note here that the deposition conditions of the 7YSZ splats were exactly the same in Figs. 2a-c and 3, as depicted in Table 1. Specifically, the consistency in deposition conditions implies the particle state (temperature and velocity) and the local surface temperature upon 7YSZ splat deposition should to be similar. The particle temperatures were measured by commercial hardware [DPV Millenium Edition, Tecnar QC Canada] to be consistent in all 7YSZ depositions. The local surface temperature upon splat impact is considered to be the same in all 7YSZ depositions by virtue of using consistent six-axis-robotically-controlled toolpaths for the depositions, and was verified to be consistent by surface pyrometry using an 8–14 μm wavelength pyrometer at a ~ 5 mm spot size on the specimen surface.

The observed deformation of the amorphous YbDS from the impact and solidification of 7YSZ clearly contrasts what is shown in Fig. 2 on stainless steel. It is agreed upon within the plasma spraying community that the traditional atmospheric deposition of molten 7YSZ droplets on metallic substrates such as steels or superalloys cannot induce remelting of the underlying substrate due to an insufficient amount of thermal energy from the impacting droplet(s)^53,60,61^. This conventional knowledge is corroborated here in this work by the FIB cross-sections of 7YSZ on stainless steel shown in Fig. 2a-c – where the splat/substrate interface remains planar and unchanged. However, it is clear in the case of Fig. 3a-d, given the change in substrate material from stainless steel to amorphous YbDS, the planarity of the interface is no longer preserved. The melting point of YbDS is around 300-400$\:^\circ\:$ C higher than the melting point of most stainless steels, thus the YbDS interfacial deformation under the same 7YSZ deposition conditions is even more surprising^62^.

One possible explanation for the unanticipated ‘softening’ and deformation of the underlying amorphous YbDS upon 7YSZ impact could be a change in viscosity of the glassy material as it locally heats due to thermal conduction transferred by the solidifying droplet. Significant viscosity changes can be possible if the amorphous YbDS approaches its glass transition temperature $\:{T}_{g}$ (even briefly). For YbDS, the glass transition temperature as it is reported in the literature appears to fall between 975 and 1025$\:^\circ\:$ C, which is obviously lower than the melting point of most steels^45,46,52^. A glass transition (or thermally) induced viscosity change and deformation of the YbDS material would have the ancillary effect of drastically changing the topography of the 7YSZ/YbDS interface, which would undoubtedly yield more mechanical interlocking and interfacial adhesion compared to a purely planar interface^63^. This could also serve to explain why there is a distinct lack of 7YSZ splat debonding in Fig. 3a-c as compared to Fig. 2c.

To prove or disprove the possibility of a temperature/glass-transition-induced softening stage, a counter experiment, wherein 7YSZ splats were sprayed onto fully crystallized YbDS, was conducted (sample C-2 in Table 1). These results are shown in Fig. 3e-h. For a similar 7YSZ splat as was studied in the previous case, there are clear differences that can be seen in the FIB cross-section. Most notable here is the lack of deformation at the 7YSZ/YbDS interface as compared to the previous specimen C-1. Obviously a fully crystallized YbDS will not undergo any glass-transition-induced ‘softening’ as heat transfers from the solidifying 7YSZ in a comparable way to the previous specimen, which suggests there is some merit to this hypothesis.

Moreover, when analyzing the specimen C-2 (Fig. 3e-h), at low magnifications it seems the 7YSZ splat is fairly adherent to the crystalline YbDS. However, higher magnification images (Fig. 3h) revealed localized debonding sites all along the 7YSZ/YbDS interface. By contrast, specimen C-1 (Fig. 3a-d), which showed much more significant YbDS deformation, showed no comparable evidence of localized debonding between 7YSZ and amorphous YbDS. These two results from Fig. 3 then complement each other, proving – from a purely mechanical perspective – there are clear adhesive advantages to be gained in spraying TBC oxides atop amorphous EBC glass-ceramics. These experimental results and accompanying analysis seem to serve as a (partial) empirical explanation as to why past works have found difficulty in directly spraying thermal barrier oxides onto pre-crystallized EBCs; from the results in this study it is clear the lack of YbDS deformation is detrimental to 7YSZ splat adherence to the YbDS EBC^43,44^.

#### YbDS migration and flow into the 7YSZ material

In addition to showing the propensity for YbDS softening, Fig. 3a-d and e-h also demonstrate important second-order consequences of a temperature/glass-transition-induced viscosity change of the underlying amorphous YbDS. From Fig. 3c and especially Fig. 3d, it is clear from the energy selective backscatter images that the amorphous YbDS material is able to flow and migrate into the 7YSZ material as it quenches. In some cases (Fig. 3c), the YbDS glass seems to ‘fill’ the gaps that would otherwise form due to microcracking events in the 7YSZ. In the larger glassy areas, such as those shown in Fig. 3c (indicated by arrows), EDS analysis within the YbDS glass material that migrated into the 7YSZ splat showed evidence of the presence of Zr. However, several micrometers deeper, in the direction of the SiC substrate, Zr was not found within the bulk YbDS EBC. More surprising is that the YbDS material is able to migrate along the boundaries of rapidly solidified 7YSZ splat grains (Fig. 3d). The length scales of these grain boundaries observed in Fig. 3d are consistent with the size of individually solidified grains or columns in 7YSZ splats as shown by Kulkarni et al. and others^54,64,65^. This phenomenon of YbDS mobility and potential Zr diffusion into the glass during 7YSZ splat solidification again was only seen when depositing onto amorphous YbDS. Furthermore, this result is consistent with the limited chemical analysis that was shown in our past work^43^. Neither migration of YbDS material nor diffusion of ionic species was observed anywhere in the pre-crystallized specimen C-2, see Fig. 3g and h.

### High-Resolution interface characterization

The FIB-SEM results in the previous section provided useful information toward the potential for enhanced mechanical adhesion due to temperature/glass-transition-induced deformations. However, the surprising success of our recent T-EBC that could withstand repeated thermal cycling might not solely be explained by enhanced mechanical interlocking^43^. As previously mentioned (and illustrated in Fig. 2), it is possible to achieve even further enhanced bonding between ceramics if and when an interexchange of chemical species takes place. Diffusion bonding in ceramics is a well-established materials processing method to achieve the upper echelon of interfacial adhesive strengths^55,57^. Yet, classical diffusion bonding is a thermally and kinetically driven process, requiring long dwell times at high temperatures. By contrast, in the plasma spray deposition process, each individual molten droplet will impact and solidify within the realm of a few microseconds (Fig. 2) – intuitively this seems to be an implausible timeframe for any diffusion bonding to occur^66,67^.

Nevertheless, the FIB-SEM-EDS results from the specimen C-1 in the prior section seemed to indicate the 7YSZ deposition caused the YbDS to soften/deform to an extent that it became mobile enough to move within both the microcrack network and nucleating grain boundaries of the 7YSZ splat during microsecond solidification. It is important to consider that from the classic diffusion bonding literature, the *rate* of formation of the diffusion bond is directly proportional to the wettability/contact area of the two ceramics^66,67^. Therefore, it could be plausible that the deformation of the amorphous YbDS during 7YSZ deposition is so extreme that it favors a diffusion bonding rate that is consistent with splat solidification i.e., these microsecond time scales. Furthermore, the results here and in our past work have suggested the possibility of Zr diffusion into the YbDS glass (and conversely, Yb diffusion into the 7YSZ), which are the prerequisite experimental indicators to determine whether or not diffusion bonding is taking place between the 7YSZ and YbDS material^43^. However, the prior characterization and even the FIB-SEM-EDS characterization presented thus far is still geometrically-limited in the sense of volumetric interactions that can skew findings. The remainder of this work aims to demonstrate through utilizing several unique higher-resolution microscopy and characterization techniques beyond traditional SEM-EDS that species interexchange does indeed take place. A combination of STEM, Laser-Assisted APT, and SEM-EBSD analyses on the T-EBC coatings that were fabricated and analyzed in our previous work will be shown in the sections that follow to prove this hypothesis^43^.

#### Characterization of the 7YSZ/YbDS interface by STEM/EDS analysis

**Fig. 4:**
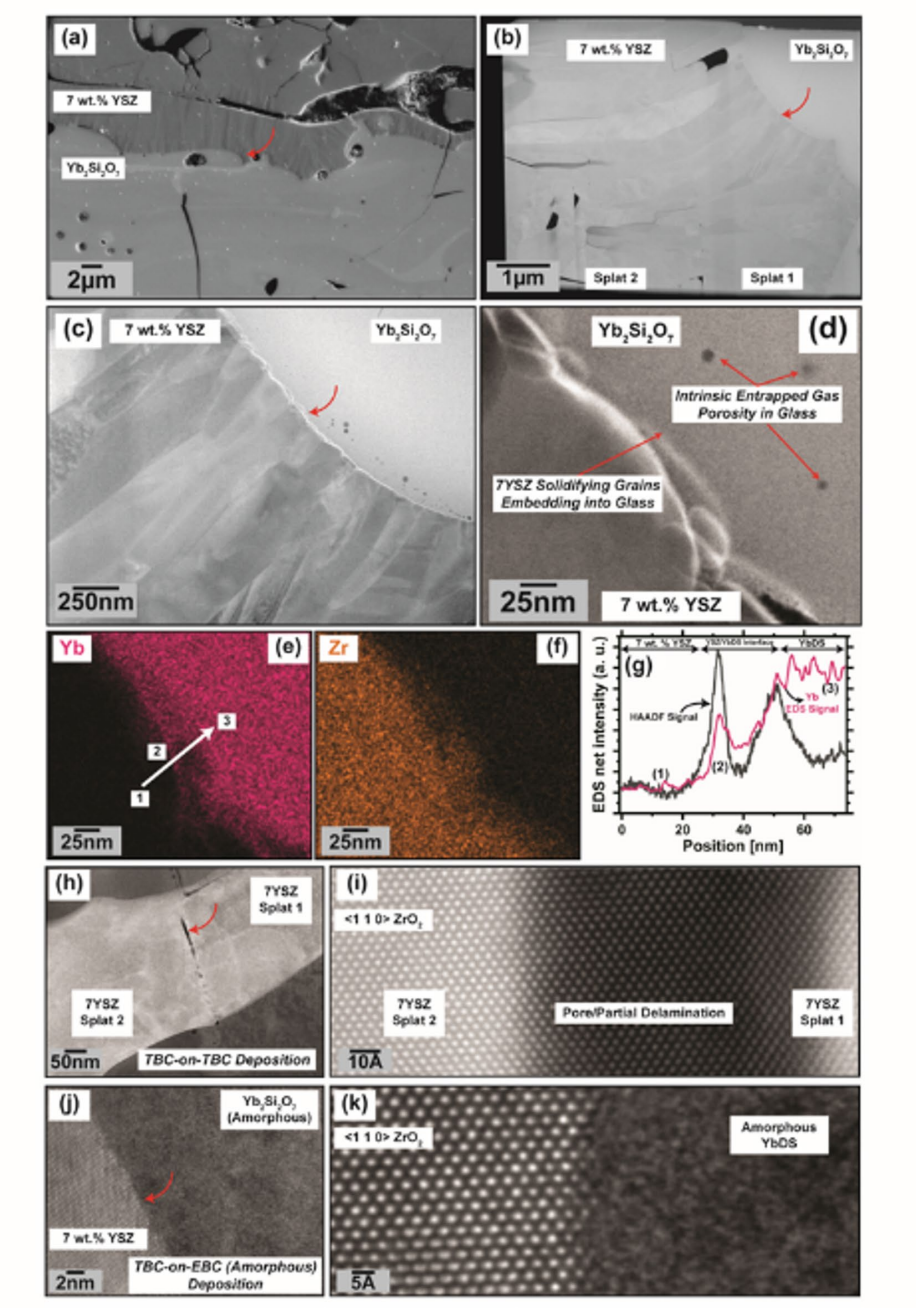
(**a**) Secondary Electron cross-section of a 7YSZ/YbDS (amorphous) as-deposited T-EBC from our past work^43^, at a location where a TEM lamella was extracted (indicated by the arrow). (**b**, **c**, **d**) HAADF images of the TEM lamella at progressively higher magnifications. The curved arrows indicate the location wherein magnification was increased for subsequent images. At the lowest magnification in (b), two 7YSZ splats can be seen. (**e**, **f**, **g**) show EDS data acquired from the location shown in (d). (**h** – **k**) are atomic-resolution images taken from the same lamella at two different locations; curved arrows indicate the region where higher-magnification images were taken for (**i**, **k**). (**h**, **i**) shows for reference the potential tendency for epitaxial growth of tetragonal ZrO_2_ as 7YSZ splats are successively deposited atop one another. The black void space in the images is clearly not a void completely through the FIB lamella, as indicated by the continuation of < 1 1 0 > reflections through the entire image in (i). (j, k) shows by contrast the impact and solidification of molten 7YSZ on amorphous YbDS does not crystallize the material.

**Fig. 5:**
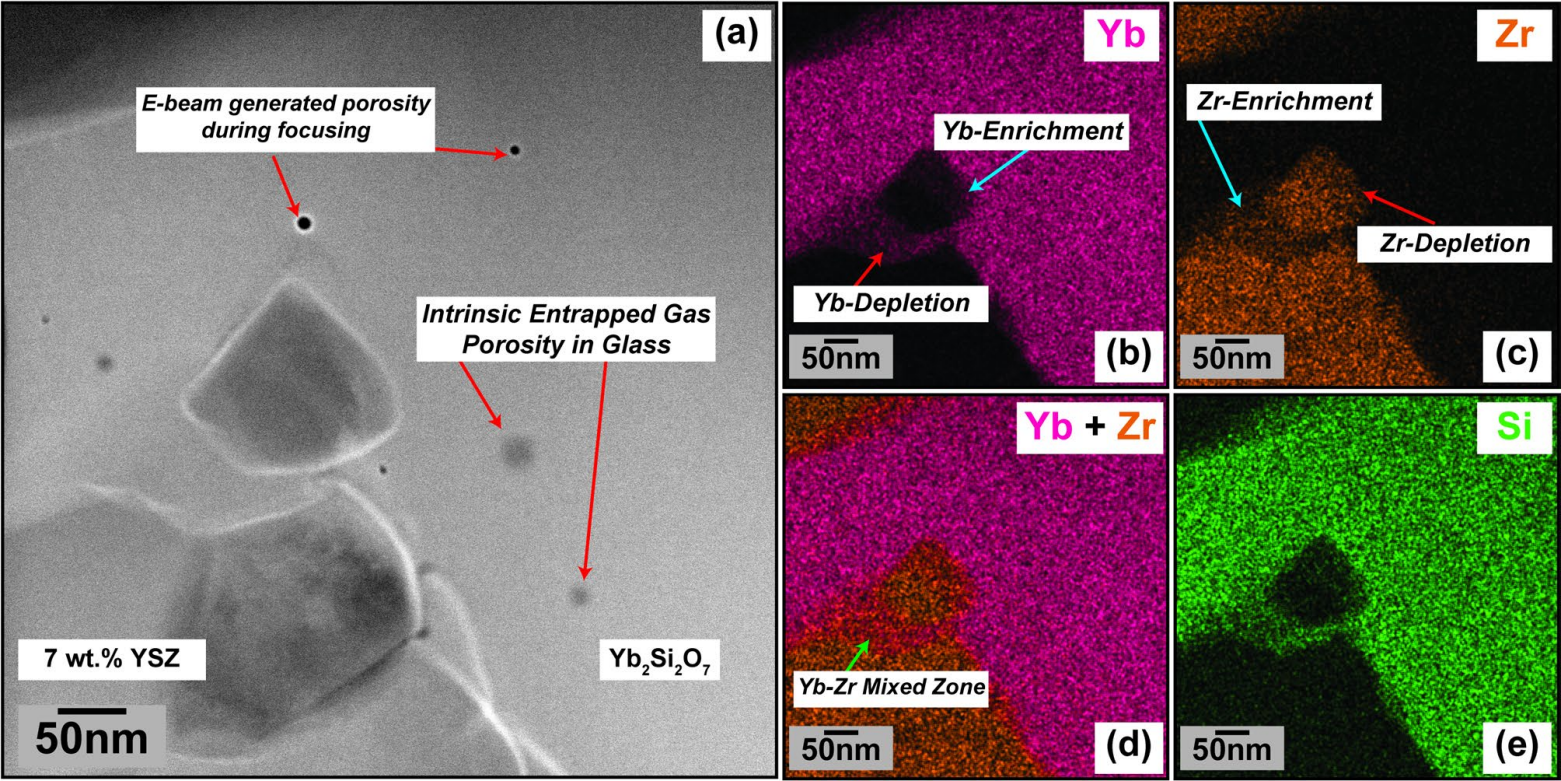
(**a**) HAADF image of the 7YSZ/YbDS STEM specimen from Fig. 4 at another location where the YbDS glass penetrates a grain boundary and seems to separate the 7YSZ material. (**b** – **e**) show EDS maps of the same location.

Figures 4 and 5 show the results of STEM imaging and high-resolution EDS analysis for an as-deposited T-EBC system (7YSZ on amorphous YbDS) from our prior work^43^. It is important to first note that when examining traditional coating cross-sections on a more macroscopic, zoomed-out scale (either Fig. 1a or Fig. 4a), the same extreme degree of YbDS interfacial deformation that was observed in Fig. 3 of the FIB-SEM results is not as obvious. In the FIB-SEM experiments, the YbDS surfaces were pre-polished to a mirror finish for optimal deposition conditions of single splats. In the case of traditional plasma spraying, this planarizing step is not done, and the EBC and TBC coatings are deposited directly atop one another with no surface pretreatments. Therefore, the deformation may be less significant when the 7YSZ impacts a naturally-rough YbDS surface. Whereas in Fig. 3 the deformations of the pre-polished YbDS were on the order of several micrometers, in Fig. 4 on the representative T-EBC the deformations are more nanometric. Consequentially, one can conclude that any YbDS/7YSZ interfacial deformation would only be able to influence the bonding of the first several 7YSZ splats that impact the amorphous YbDS surface.

Nevertheless, as can be seen in the higher-magnification HAADF images (Figs. 4c and d and 5a), there is still clearly some physical deformation phenomena occurring at the sub-micro or nano scale as the first 7YSZ splats impact the amorphous YbDS. In the case of this STEM specimen, the individual grains or columns within the 7YSZ splat appear to have ‘embedded’ themselves within the glassy YbDS material (Fig. 4d, arrow; Fig. 5a). Furthermore, the same ‘glassy mobility’ from Fig. 3c and d that should enable YbDS penetration into the 7YSZ grains/grain boundaries during solidification is clearly seen in Fig. 5a, in this case even allowing complete separation of a single 7YSZ grain from the rest of the splat material. These STEM results are then consistent with what was observed in the FIB-SEM experiments in the prior section in Fig. 3d. A logical counterpoint to the hypothesis of viscosity-induced softening would be if the impact and solidification of $\:>$2700$\:^\circ\:$C molten 7YSZ onto glassy YbDS inspires crystallization of the glass. The atomic resolution images in Fig. 4h-k serve to provide essential information toward addressing this counterpoint.

The results shown in Fig. 4h-k strongly suggest that the impact and solidification of molten 7YSZ splats does not create a condition wherein the YbDS can locally crystallize. This becomes especially clear in Fig. 4k, where there is absolutely no evidence of atomic order in the YbDS glass (even on angstrom-length-scales near the 7YSZ splat). Therefore, if there is absolutely no crystallization occurring, then a localized viscosity change with elevated YbDS temperature upon molten 7YSZ impact is highly plausible.

Both Figs. 4 and 5 present corroborating evidence supporting the species interexchange hypothesis. First, in Fig. 4d, the bright features at the base of the 7YSZ columns in the HAADF images were determined by EDS to be Yb-rich (and somewhat Zr-lean) regions. This becomes clear when cross-examining the EDS maps against the linescan plot in Fig. 4e, f, and g, respectively. At Point 2 on the EDS map in Fig. 4e, the HAADF and Yb signal are both indicating a local maximum (Fig. 4g). Likewise in Fig. 5, in the region where the glassy YbDS separated the 7YSZ grain from the rest of the splat, the EDS maps show there is a noticeable concentration gradient in both Yb and Zr surrounding the 7YSZ grain (Fig. 5b and c). Despite these additional insights and supportive evidence toward interdiffusion gained from this higher-resolution-EDS, there are still geometrical caveats and limitations to the interpretations to consider. Therefore, a complimentary and altogether unique technique of Laser Assisted Atom Probe Tomography (LAAPT) was used to study the chemical species and their positions at the same 7YSZ/YbDS interface shown in Fig. 4a.

#### Laser-assisted APT characterization of the interface

**Fig. 6:**
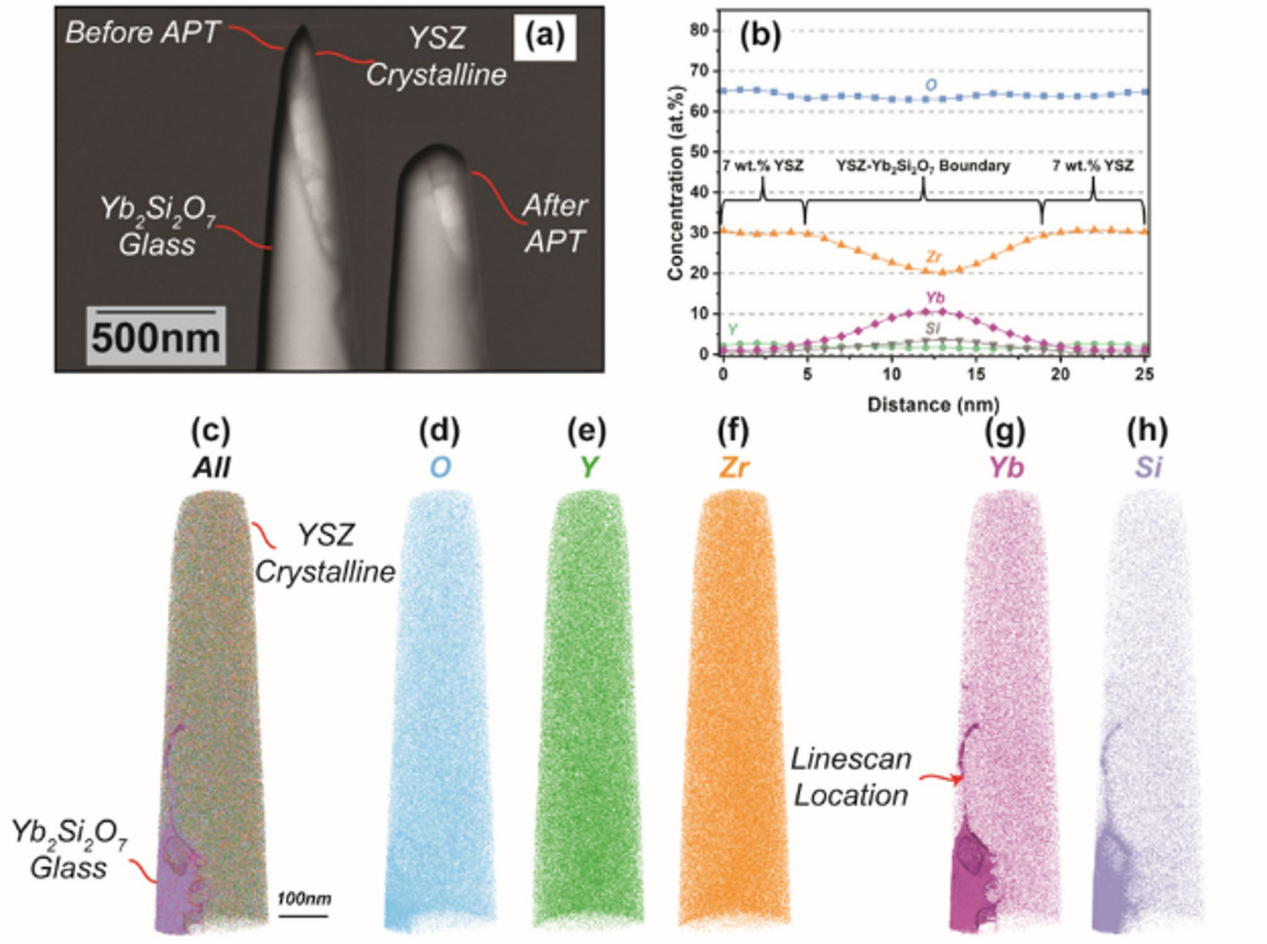
Laser Assisted Atom Probe Tomography (LAAPT) results for the 7YSZ/YbDS interface at the location shown in Fig. 4a. (**a**) shows secondary electron images of the FIB-extracted APT needle before and after measurement. (**b**) shows a concentration profile taken normal to a region of Yb-rich migration, the location of which is indicated by an arrow. (**c-h**) show 3-dimensional renderings of the dataset and their respective positions within the needle.

Figure 6 shows the LAAPT data generated from the same location as indicated in Fig. 4a. A ‘needle’ at this location was extracted by FIB-milling, as described in the experimental section. In Atom Probe Tomography, an ablation source (either electric field in the case of conductive samples, or laser in the case of insulative samples) impinges on the cryogenically-cooled needle specimen – eliciting localized evaporation in a vacuum chamber. A mass spectrometer then gathers and collects the incident species and interpolates the 3-Dimensional positioning of each atom^68,69^. Thus, Fig. 6c-h are 3-Dimensional renderings of the ionic species present in the evaporated portion of the needle shown in Fig. 6a. A concentration linescan (Fig. 6b) orthogonal to the vertically-protruding YbDS material, located at the arrow in Fig. 6g was extracted. Most notably, the data obtained in Fig. 6 supports two key observations from the earlier experiments: first, there is again evidence that the amorphous YbDS material is mobile enough to ‘flow’ within the 7YSZ material (Fig. 6g, h). Second, in areas of contact between 7YSZ and amorphous YbDS, there is clearly a nanoscale concentration gradient of Yb and Zr, which indicates species interexchange occurred during 7YSZ deposition (Fig. 6b).

**Table 2:** Atomic concentrations of ions taken from within either the bulk Yb_2_Si_2_O_7_ glassy phase or the bulk 7 wt% YSZ ceramic phase in the LAAPT specimen.

Bulk Yb_2_Si_2_O_7_		Bulk 7YSZ	
**Ion**	**Concentration (at%)**	**Ion**	**Concentration (at%)**
O	57.0 $\:\pm\:$ 0.10	O	67.2 $\:\pm\:$ 0.01
Yb	25.0 $\:\pm\:$ 0.12	Yb	0.01 $\:\pm\:$ 0.01
Si	14.0 $\:\pm\:$ 0.10	Si	0.024 $\:\pm\:$ 0.004
Zr	3.54 $\:\pm\:$ 0.03	Zr	29.9 $\:\pm\:$ 0.01
Y	0.275 $\:\pm\:$ 0.01	Y	2.54 $\:\pm\:$ 0.007

To fully appreciate the concentration gradients shown in Fig. 6b, concentration analyses within the predominantly 7YSZ and YbDS regions of the APT datasets were conducted. The quantification results of this analysis are shown in Table 2. Here these regions are defined as ‘bulk’ concentrations, although it is clear from the Table that the available YbDS region analyzed in this dataset still kept some YSZ within itself.

When comparing Table 2 with Fig. 6b, two key conclusions can be drawn. First, it is clear that directly to the left and right hand side of the protruding YbDS line in Fig. 6g, h, there is little to no YbDS present – implying there was a preferential pathway for the glassy phase to move within the 7YSZ splat. Morphologically, the migration pathway of the YbDS here appears to be along a 7YSZ grain boundary (made visible only by the material contrast in the APT rendering), which is consistent with the FIB-SEM results.

The concentration of Zr at these left and rightmost regions in Fig. 6b are consistent with the bulk Zr concentrations of 7YSZ found in Table 2 (in both locations, ~ 30 at%). Additionally, as the linescan moves through the YbDS protrusion, it is clear the Zr concentration depletes and the Yb concentration increases. However, neither ionic concentration reaches its bulk value as indicated in Table 2. This further suggests an interexchange between Yb and Zr took place in this migrated region where the YbDS glass was mobile, and new equilibrium concentrations were found. Given the ultrahigh spatial and concentration resolution of APT, these concentration values can be considered to be highly accurate and irrefutably indicate the presence of both Yb and Zr in the glass^68,69^. The local minimum 20 at% and maximum 10 at% concentrations of Zr and Yb, respectively were then chosen to generate isocontour maps at the YbDS-rich region of the dataset toward the bottom of the needle (seen in Fig. 6c).

**Fig. 7:**
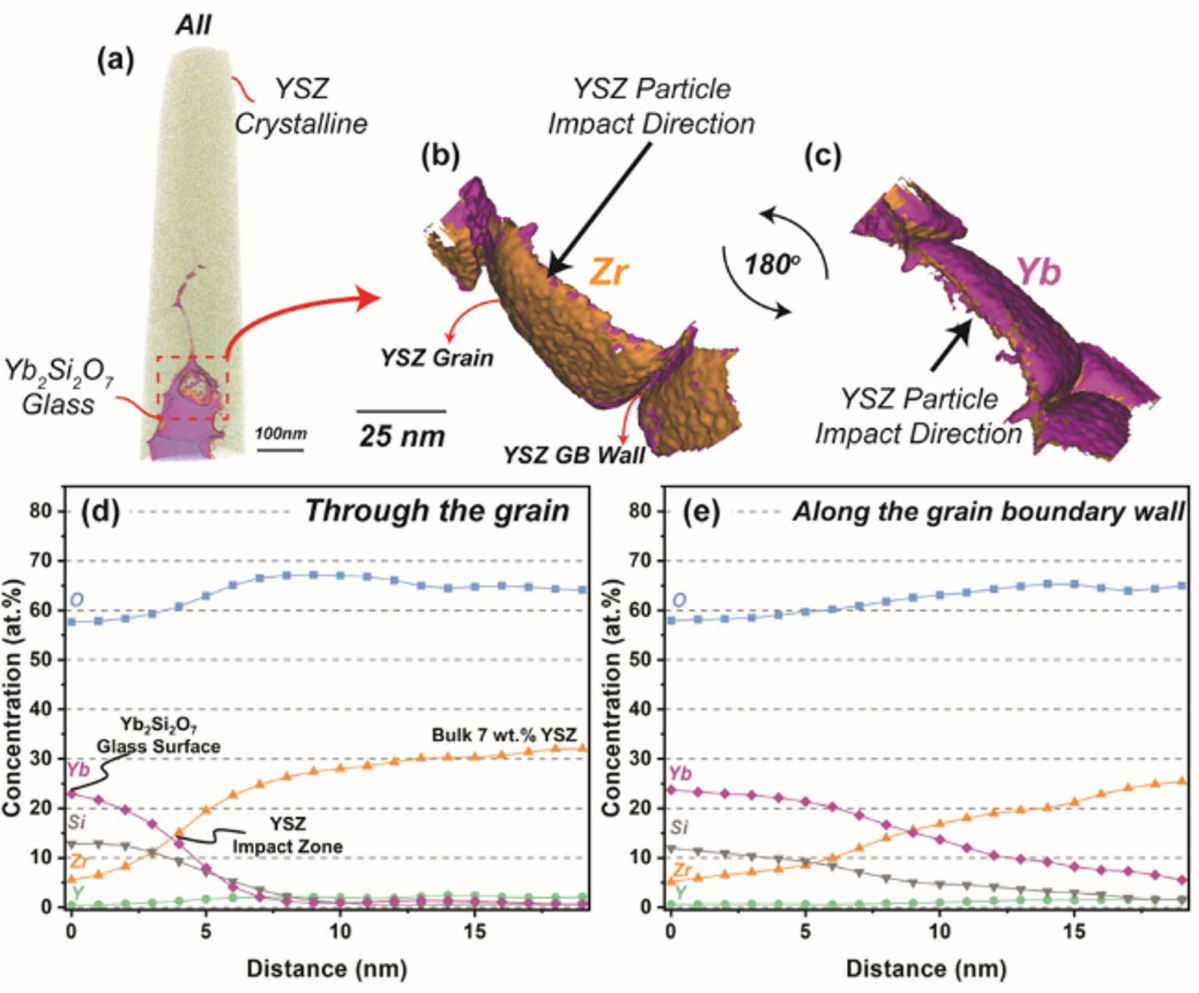
(**a**) 3-dimensional rendering of the original APT dataset from Fig. 6, rotated to put the YbDS glass in the field of view. (**b**, **c**) Isocontours of Zr at 20 at% and Yb at 10 at% at the location boxed in (a), the direction of incoming 7YSZ particles during plasma spray deposition is illustrated with an arrow. (**d**) atomic concentration profile through the large grain in the center of (b, c). (**e**) atomic concentration profile through a grain boundary from (b, c). Both atomic concentration profiles begin at the YbDS bulk glass (x = 0 nm) and travel into the YSZ material (x = 20 nm).

Figure 7 shows the aforementioned isocontour maps derived from the location in the APT needle where the YbDS glass is most prominent (indicated by a box in Fig. 7a), as well as two more concentration linescans. From the maps in Fig. 7b and c, there are morphological consistencies with what was seen in both the FIB-SEM and STEM experiments. Again, in Fig. 7, there is clear evidence that the grains of solidifying 7YSZ material have a tendency to deform or ‘embed’ themselves within the YbDS glass. It is important to note the 7YSZ material comes *after* the amorphous YbDS is already deposited (the impact direction of the 7YSZ droplets is shown in the Figure). Moreover, it is obvious from Fig. 7b and c that the 7YSZ grains not only embed themselves within the YbDS glass, but also cause ‘flow’ or ‘protrusions’ of the glassy material along the developing 7YSZ grain boundaries.

Two concentration linescans were conducted: through the center of the 7YSZ grain and along the grain boundary and are shown in Fig. 7d and e. Clearly, there is species interexchange at the nanoscale that not only takes place at the contact points of the 7YSZ grains with the YbDS glass, but also along the 7YSZ grain boundaries themselves. In essence, it can be said that during 7YSZ deposition, wherever the glassy YbDS came into contact with the 7YSZ material, species interexchange occurred. This is even more obvious when examining Fig. 7e, where the glassy YbDS is seen to be in contact with 7YSZ for much longer distances, as it flows along the boundary between two grains. It is possible this is a consequence of increased capillary forces at such narrow opening dimensions which are able to drive glass infiltration at these length scales^70^. Furthermore, it can be seen in this particular linescan along the grain boundary that the Yb concentration never drops to absolute zero, whereas in the linescan through the grain (Fig. 7d), it nearly does.

### Proposed mechanism for enhanced interlayer adhesion in an effective T-EBC

From these results, in conjunction with what we observed in our past work^43^, it can be concluded that the high durability of the 7YSZ/amorphous YbDS T-EBCs is a consequence of several concurrent phenomena that are unique to spraying onto an amorphous glassy-ceramic material. The mechanism that is hypothesized to yield highly adherent 7YSZ coatings atop CTE-mismatched YbDS/Si/SiC systems is a two-stage mechanism that evolves over time and temperature, starting with the onset of nanoscale diffusion that takes place during deposition and rapid solidification, and is described below.

#### Stage 1 – Interdiffusion of yb, Zr upon deposition of 7YSZ onto amorphous YbDS

When examining all the FIB-SEM, STEM, and LAAPT findings concurrently, one can interpret the probable temperature evolution at this interface as molten (> 2700$\:^\circ\:$C) 7YSZ impacts warm (~ 300-400$\:^\circ\:$ C) amorphous YbDS. The FIB-SEM experiments, have shown the YSZ splats are able to deform, interact with, and distort the YbDS glass (Fig. 3). Additionally, the STEM has shown that crystallization upon impact does not occur (Fig. 4i). From the literature, it is known that crystallization of YbDS begins around 1050$\:^\circ\:$C^46,52^. The distortion of the glass suggests that the 7YSZ deposition process (on these short microsecond time scales) is indeed able to raise the temperature of YbDS quite significantly. However, the kinetics during 7YSZ splat deposition are clearly insufficient to crystallize the YbDS. Past works have shown the crystallization of APS YbDS to be (relative to microsecond timescales where deformation and interdiffusion can take place) rather sluggish – requiring the formation/transformation of metastable YbDS and YbMS phases before full crystallization is realized, which supports this hypothesis^52,71^. Therefore, it would appear more likely that the YbDS is brought to a temperature close to its glass transition temperature $\:{T}_{g}$. As the $\:{T}_{g}$ is approached, the viscosity of the YbDS should reduce dramatically, enhancing material mobility^46^.

Consequentially, the YbDS viscosity/mobility change would thereby allow the glass to migrate into the cracks of the 7YSZ splat(s) and even (as has been shown in the results i.e., Fig. 3) along the splat grain boundaries^46,72,73^. Examining the STEM, EDS, and APT results concurrently has suggested that the free migration of glassy YbDS material during deposition of 7YSZ facilitates markedly enhanced physical contact between the dissimilar ceramics. Consequentially, the intimate material contact favors interexchange of ionic species is favored during 7YSZ quenching. The Yb (and to some extent the Si) from the YbDS seems to migrate into the ZrO_2_ lattice of the YSZ; and conversely, there is Zr (and to some extent Y) migration into the YbDS glass. The concurrent interexchange of ionic species during deposition was not observed to influence or change the overall phase/structure of the 7YSZ (Fig. 4h). This allows the 7YSZ splats at this interface to keep their advantageous high-toughness tetragonal structure after TBC deposition is complete, thereby eliminating the possibility of the formation of a weak interface due to ZrO_2_ phase change^74^.

#### Stage 2 – Evolution of the interface over prolonged thermal exposure

**Fig. 8:**
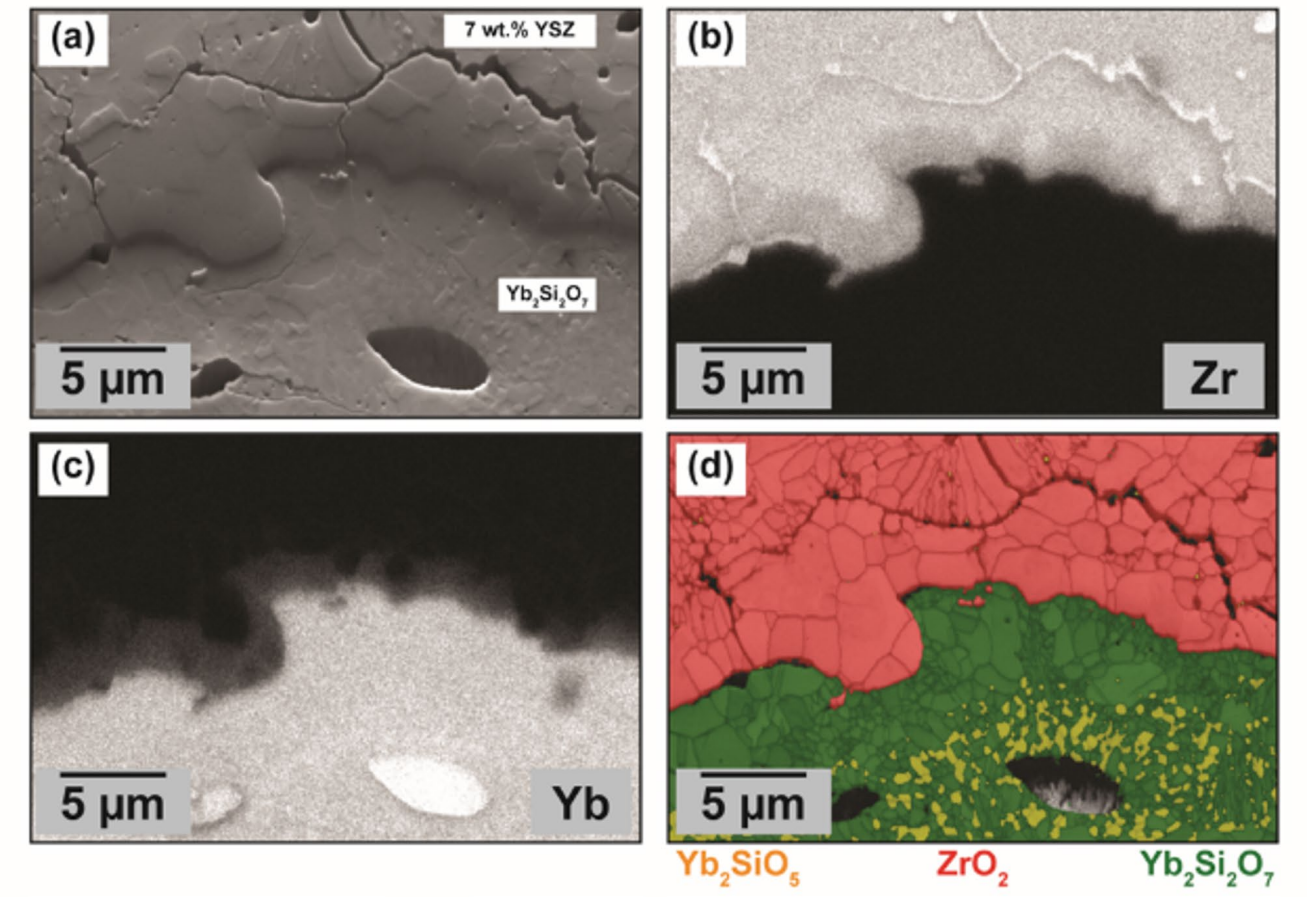
(**a**) Forward scattered electron (FSE) image of the 7YSZ/YbDS interface in a T-EBC sample from our past work after 200 hours’ thermal treatment at 1300$\:^\circ\:$C^43^. (**b**) Zr EDS map of the same location. (**c**) Yb map of the same location. (**d**) EBSD band contrast map of the same location, indicating the phases of < 1 1 0 > tetragonal ZrO_2_, Yb_2_SiO_5_, and Yb_2_Si_2_O_7_.

Over prolonged thermal exposure (for instance at 1300$\:^\circ\:$C over several hundred hours) the species interexchange that is now proven to take place upon 7YSZ deposition continues to progress in a steady manner (shown in Fig. 1b and in our past work)^43^. Figure 8 shows higher-resolution SEM, EDS, and EBSD images of such a thermally-treated T-EBC from our past work which had undergone 200 hours’ thermal exposure at 1300$\:^\circ\:$C^43^. Clearly from the EDS maps in Fig. 8b and c, the prolonged thermal exposure has facilitated Yb enrichment and Zr depletion at the 7YSZ/YbDS interface to much larger length scales than what the FIB-SEM, STEM, and LAAPT had shown in the prior sections. Likewise, in agreement with the STEM results from Fig. 4i, the continued interdiffusion of Yb and Zr appears from the EBSD mapping to have no influence on the tetragonality of the ZrO_2_ phase in the 7YSZ near the interface – suggesting no depreciation in intrinsic toughness from the as-sprayed or virgin state^74,75^.

In such a case, as the interface evolves and interdiffusion continues, as illustrated in Fig. 2, its adhesive strength and toughness should far exceed what is possible by conventional mechanical interlocking. This work has proven that while there is undeniable softening and deformation of the amorphous YbDS upon molten 7YSZ impact, the topographical changes in a real T-EBC are constrained to the sub-micrometer scale. As such, these small topography changes are unlikely to have a substantial mechanical influence on the overall adherence of the 7YSZ layer atop the YbDS/Si/SiC base material. However, the deformation is assumed to work concurrently with enhanced glass mobility as a prerequisite to facilitate an otherwise unattainable degree of 7YSZ/YbDS contact area that would inspire enhanced rates of interdiffusion (both intragranular and along grain boundaries). As this now-diffusion-bonded 7YSZ/YbDS interface then continues to evolve, diffuse, and grow in length over prolonged thermal exposure, the imparted adhesive benefits are surmisable to outweigh the large CTE-mismatch-driven stresses that arise at this location during each thermal cycle.

The retention of the high-toughness < 1 1 0 > tetragonal ZrO_2_ phase over continued thermal exposure and diffusion would also further explain how the high-CTE 7YSZ can resist fracture and remain adherent to a low-CTE YbDS/Si/SiC structure after deposition and after repeated thermal cycling. Clearly from these results, an optimal T-EBC is one which is fabricated utilizing this deformation-enhanced interdiffusion to form an undeniably adherent (and tough) diffusion-bonded interface between the oxide and glass-ceramic.

## Conclusions

This work has shown, through the synthesis of results from four independent characterization techniques, the underlying mechanisms that take place during 7YSZ deposition atop ytterbium silicate ceramics of varying crystallinity. By revealing these brand-new mechanisms, a strategy toward how the plasma spraying process can be tailored to facilitate successful fabrication of adherent, durable Thermal-Environmental Barrier Coatings for future ultrahigh temperature, energy-efficient gas turbine engines has been reconciled.

One of the most salient findings from this work is the influence of the crystallinity of the underlying EBC ceramic on governing mechanisms of interfacial bonding prior to TBC deposition. It is now clear that when TBC oxides are sprayed atop amorphous glassy silicate ceramics, advantageous thermomechanical and thermochemical interactions take place. The impact and rapid solidification of molten 7YSZ atop amorphous ytterbium disilicate is clearly shown to – even on these short microsecond timescales – achieve the necessary conditions to raise the silicate at or above its glass transition temperature. During this time and temperature increase, the silicate material undergoes substantial viscosity changes, which allow it to not only deform and contort to the structure of the quenching 7YSZ splat, but also increases the mobility of the glass – allowing glass infiltration within the grain boundaries of the 7YSZ that form during solidification, thereby enhancing overall 7YSZ/YbDS contact.

It was shown that in spite of these locally intense thermal events, the timescales of 7YSZ splat deposition were far too short to induce any type of localized crystallization of the amorphous ytterbium silicate layer. Nonetheless, as the glassy ceramic comes into intimate contact with the solidifying 7YSZ material, interexchange of species takes place on the nanoscale. From there, a diffusion-bonded interface forms wherever there is contact between 7YSZ/YbDS. The inevitable subsequent thermal exposures after T-EBC fabrication (i.e., for final crystallization of the inner EBC layer, thermal cycling during operation, etc.) can further facilitate interdiffusion and a clear reaction zone forms. Neither during 7YSZ splat deposition nor after prolonged thermal exposure does the 7YSZ structure at this interface change from its advantageous high-toughness tetragonal phase. None of these advantageous phenomena take place when 7YSZ is deposited atop pre-crystallized silicates, which cannot undergo viscosity-induced softening and deformation during deposition. This can serve to explain why in previously published research it has been incredibly challenging to successfully fabricate T-EBCs – because these previously published efforts were focused on depositing atop pre-crystallized EBCs. The results from this work should encourage future study of T-EBCs that can be successfully deposited and studied for their thermomechanical durability.

## Data Availability

The datasets used and/or analysed during the current study available from the corresponding author on reasonable request.
